# Usage of smart devices amongst medical practitioners in Universitas Academic Hospital

**DOI:** 10.4102/safp.v62i1.5029

**Published:** 2020-02-04

**Authors:** Yeyang Xu, Zoë Francis, Khayam Saleem, Siphamandla Sambujana, Keitumetse Molise, Boitumelo Molise, Nicholas Pearce, Gina Joubert

**Affiliations:** 1 Department of Surgery, Faculty of Health Sciences, University of the Free State, Bloemfontein, South Africa; 2 Department of Biostatistics, Faculty of Health Sciences, University of the Free State, Bloemfontein, South Africa

**Keywords:** smartphones, confidentiality, patient preference, usage, medical practitioners

## Abstract

**Background:**

There has been a rapid rise in the use of smart devices amongst medical practitioners throughout the world. This study aimed to identify how smart devices were being used by medical practitioners at the Universitas Academic Hospital (UAH), Bloemfontein, and the associated factors thereof. We also identified the views of medical practitioners regarding the usage of smart devices at their workplace.

**Methods:**

A prospective cross-sectional study was conducted. Anonymous questionnaires were distributed to medical practitioners working at UAH during weekly departmental meetings or monthly morbidity and mortality meetings. The following largest departments were included: Surgery, Anaesthetics, Paediatrics, Internal Medicine, Family Medicine, and Obstetrics and Gynaecology.

**Results:**

The response rate was 82.7% of those attending the meetings. All the respondents owned a smart device and brought it to their workplace. The most common applications used on these smart devices were that for drug references (65.9%), medical textbooks (63.6%) and medical calculators (58.1%). Significantly larger percentages of doctors aged 21–39 years compared with those aged 40–65 years used drug reference applications and medical calculators. A quarter (24.8%) of respondents communicated with patients through a smart device, 21.7% used an online storage platform to backup patient data, whilst 56.6% used their devices to store and view patient information. More than one-third (36.7%) agreed that smart devices threatened patient confidentiality, but the majority (58.8%) did not agree that these devices hinder patient communication. The majority felt that these devices improved both personal performance (69.2%) and patient care (79.0%).

**Conclusion:**

Smart devices usage is common in this setting. Hence, integration of such usage in medical curricula, discussion on professionalism, ethics and confidentiality in this context, and guidance from institutions and professional bodies become necessary.

## Introduction

There has been a rapid rise in the use of smart devices (mobile devices such as tablets, laptops and cell phones) amongst medical practitioners throughout the world.^1,2,3,4^ As a result, many applications have been developed to suit this rise and need. A systematic review showed that the applications developed include the ones which assist healthcare professionals with patient data management and those that serve as reference or training for healthcare professionals.^1^ Because of the diverse number of applications, there have been concerns regarding the quality of information.^5^ Applications for patients themselves include the applications which support patients in chronic disease management and the ones that allow them to communicate with medical practitioners.^1^

Communication between different staff within a hospital has changed considerably.^6^ In the 1990s, one would almost solely rely on pagers as the mode of connection between medical practitioners and other staff members within a hospital, but now pagers are replaced by smart devices or personal digital assistants as mode of communication.^6^

Progress in technology has made the transfer of information easier. Improvement in the quality of mobile cameras and speed of the Internet have allowed the sending of patient X-rays, computerized tomography (CT) scans and other images and videos much more conveniently.^4,7^ Unlike pagers, smart devices provide various platforms of communication such as WhatsApp, emails, phone calls and text messages. Rapid data entry is achieved with the built-in keyboards on these devices.^6^

Confidentiality of patient information is of concern because medical practitioners could easily distribute and share this information.^2,8^ WhatsApp and other social media platforms allow medical practitioners to share images and other diagnostic information with colleagues for diagnoses, but at the same time, this information could be seen by people who are not permitted to view the data, thus compromising patient confidentiality.^8,9^

Limited research has been published on how medical practitioners in South Africa use their smart devices for professional purposes. We identified only two South African reports, but they did not match the context with which this study has been initiated.^9,10^ The first study investigated the impact of telecommunication on the management of burn wound patients.^9^ The study concluded that the implementation of telemedicine saved the cost of transfer and improved patient care by making more accurate referrals. However, the issue of confidentially of images needs to be addressed. The second, an editorial, focused on whether smart devices improved performance of nurses as it allows instant access to a clinical library and treatment guidelines.^10^ Studies elsewhere have investigated usage of smart devices in specific disciplines^3,5,9,11^ or amongst medical students, residents and junior doctors.^12,13,14,15,16,17^

## Aim

The aim of this study was to identify the ways smart devices are being used by medical practitioners at the Universitas Academic Hospital (UAH), Bloemfontein, and the associated factors thereof.

Specific objectives:

- To identify the types of smart devices used for work purposes at the hospital.
- To determine which medical applications are there on the smart devices of medical practitioners.
- To identify the work-related activities for which smart devices are used by medical practitioners.
- To identify the views of medical practitioners regarding the use of smart devices at their workplace.
- To identify whether age plays a role in the use of medical applications.
- To identify the difference in the use of smart devices between different departments.

## Method

This was a prospective analytical cross-sectional study. The study population comprised medical practitioners working in the largest departments of UAH, namely, Departments of Surgery, Anaesthetics, Paediatrics, Internal Medicine, Family Medicine, and Obstetrics and Gynaecology. The inclusion criteria being the head of department or unit, consultants, registrars and interns in the age group 21–65 years.

### Measurements

Arrangements were made with the head of each department, and a convenient day was fixed for data collection using an anonymous and self-administered questionnaire. The questionnaire was compiled by the authors using related questionnaires^3,11,12,13,14,15^ obtained from the literature. Questionnaires were distributed for immediate completion at departmental as well as morbidity and mortality meetings of each selected department. Respondents who had previously completed the questionnaire were requested not to fill these again. The questionnaire included questions about their rank, age category, department and type of smart device used by respondents. The questionnaire was in English only.

### Pilot study

The pilot study was performed on 10 medical practitioners from the Department of Orthopaedics. Subsequent changes made to the questionnaire included removing some of the questions and correcting spellings and grammatical errors. The pilot study results were excluded from the main study.

### Data analysis

The data were coded and entered into an Excel spreadsheet. Data analysis was performed by the Department of Biostatistics, Faculty of Health Sciences at the University of the Free State (UFS) using SAS software, version 9.3. Results are summarised by frequencies and percentages. Denominators are indicated throughout because some respondents did not complete all the questions. Subgroups were compared using chi-square or Fisher’s exact tests.

### Ethical considerations

Each respondent received an information leaflet about the aim of the study. By completing the anonymous questionnaire, respondents agreed to participate in the study. Participation in the study was voluntary and respondents were free to withdraw from the study at any time.

The protocol was approved by the Health Sciences Research Ethics Committee, UFS (HSREC-S 15/2017), and the Free State Department of Health gave permission to conduct the study.

## Results

Of the 156 medical practitioners who attended various meetings, 131 completed the questionnaire. Two questionnaires were excluded because their respondents did not comply with the inclusion criteria; hence, 129 questionnaires were included in the study (response rate 82.7% of those who attended various meetings).

Most of the respondents (80.6%) were in the age group 21–39 years (Table 1). The highest percentage of respondents was either head of departments/units or consultants (34.1%) and interns (34.1%). Half of the respondents were from the Departments of Surgery (30.2%) and Internal Medicine (23.3%).

**TABLE 1:** Demographic information of participating medical practitioners.

Variable	*n*	%
**Age (*n* = 124)**		
21–39	100	80.6
40–65	24	19.4
**Rank (*n* = 126)**		
Head of department/unit or consultant	43	34.1
Registrar	37	29.4
Intern	43	34.1
Other	3	2.4
**Department (*n* = 129)**		
Surgery	39	30.2
Internal Medicine	30	23.3
Obstetrics and Gynaecology	20	15.5
Family Medicine	19	14.7
Paediatrics	13	10.1
Anaesthetics	8	6.2

All the respondents (*n* = 129) indicated that they had smart devices which they carried to work. Almost all of the respondents brought their cell phones (*n* = 127, 98.5%), whilst 43.4% (*n* = 56) brought their tablets and 33.3% (*n* = 43) their laptops. The types of medical applications uploaded to these devices and work-related usage of these smart devices are listed in Table 2.

**TABLE 2:** Applications on smart devices and work-related usage (*n* = 129).

Variable	*n*	%
**Type of medical application**		
Drug reference	85	65.9
Medical textbooks	82	63.6
Medical calculator	75	58.1
Literature search	60	46.5
Disease diagnosis	56	43.4
Medical training	33	25.6
Patient communication	16	12.4
Patient education	9	7.0
Clinical communication	11	8.5
**Work-related usage**		
Colleague communication	112	86.8
Storage and usage of medical books	92	71.3
Patient information storage and viewing	73	56.6
Patient communication	32	24.8
Online backup of patient information	28	21.7

More than half of the respondents had applications for drug reference (65.9%), medical textbooks (63.6%) or a medical calculator (58.1%) on their smart devices. Few respondents had an application for clinical communication (8.5%) or patient education (7.0%). Respondents mostly used their smart devices for communication with colleagues (86.8%) or storage and use of medical textbooks (71.3%). The median number of types of applications was three (range 0–9).

When comparing the age groups 21–39 years and 40–65 years regarding medical applications, statistically, a significantly larger percentage of younger age group had drug reference applications (72.0% vs. 45.8%, *p* = 0.03) and a medical calculator (63.0% vs. 37.5%, *p* = 0.01). Median numbers of 4 and 2 were obtained for the type of applications in the younger age group and the older age group, respectively (*p* = 0.04). As far as work-related usage is concerned, statistically, a significantly larger percentage of respondents in the age group 21–39 years used smart devices for storage and usage of medical textbooks (78.0% vs. 45.8%, *p* < 0.01).

Departments of Surgery and Internal Medicine, the two largest departments, were compared in a similar manner. Statistically, there was a significant difference between the two departments regarding having an application to communicate with patients (21.1% vs. 0.0%, *p* = 0.01). The median number for the types of applications was 3 in Surgery and 4 in Internal Medicine, respectively. Statistically, there were no significant differences between the two departments about the usage of their smart devices for any of the work-related activities. In both these departments, the median age of participants was 32 years. In the Department of Surgery, the largest percentage of respondents was registrars (40.5%) whereas in the Department of Internal Medicine, the largest percentage of respondents was consultants (38.7%).

Table 3 provides the frequency of different work-related activities performed by medical practitioners on their smart devices.

**TABLE 3:** Frequency of work-related activities performed on their smart devices.

Question	Always†		Sometimes†		Occasionally†		Never†	
	*n*	%	*n*	%	*n*	%	*n*	%
**Information handling**								
I record patient information on my smart device	13	10.1	34	26.4	48	37.2	34	26.4
I use my smart device to send information to other doctors	38	29.5	57	44.2	30	23.3	4	3.1
I use WhatsApp to send images of patients for opinions	29	22.5	54	41.9	29	22.5	17	13.2
I use my device to email patient information	2	1.6	12	9.3	41	31.8	74	57.4
**Social media**								
I look up images of patients on social media (*n* = 126)	3	2.4	6	4.8	15	11.9	102	81.0
I post images of patients on social media (*n* = 127)	2	1.6	1	0.8	5	3.9	119	93.7
**Diagnosis**								
I look up diagnoses on Google	10	7.8	48	37.2	50	38.8	21	16.3
I look up diagnoses on medical apps (*n* = 128)	23	18.0	36	28.1	54	42.2	15	11.7
**Drug reference**								
I look up drug dosages	29	22.5	67	51.9	26	20.2	7	5.4
I use my device to look up drug interactions	19	14.7	62	48.1	41	31.8	7	5.4
**Administrative work**								
I manage patient admissions/operations/investigations etc. (*n* = 128)	8	6.3	28	21.9	26	20.3	66	51.6
I maintain a logbook on my smart device	17	13.2	6	4.7	17	13.2	89	69.0
**References**								
I use medical calculators to assist me in working	10	7.8	46	35.7	45	34.9	28	21.7
I look up anatomy on my device	10	7.8	31	24.0	50	38.8	38	29.5
I look up diagnostic criteria	13	10.1	62	48.1	42	32.6	12	9.3
I use my device to look up procedure codes (ICD10, CPT4, etc.)	38	29.5	28	21.7	34	26.4	29	22.5
**Private communication**								
I answer private calls whilst with a patient (*n* = 128)	2	1.6	12	9.4	55	43.0	59	46.1
I sms/WhatsApp whilst interacting with a patient	0	0	9	7.0	53	41.1	67	51.9
**Emergency care**								
I use my smart device for emergency care (*n* = 119)	9	7.6	40	33.6	53	44.5	17	14.3

Note: All *n* = 129 unless indicated otherwise.

† , ‘always’ = every day or second day, ‘sometimes’ = once or twice a week, ‘occasionally’ = a few times a month.

Regarding information handling and administrative work, 37.2% of the respondents occasionally recorded patient information on their smart device and sometimes send information (44.2%) or images (41.9%) to other doctors. Only a few respondents reported that they always looked at (2.4%) or posted (1.6%) images of patients on social media. Half of the respondents (51.6%) never managed patient admissions or operations through their smart device, whilst 69.0% never maintained a logbook on their device.

Respondents occasionally referred to Google for diagnosis (38.8%) or medical application (42.2%). Half of the respondents used their smart device sometimes to look for drug dosages (51.9%) and drug interactions (48.1%), whilst 44.5% occasionally used their smart device for emergency care.

Regarding private communication, approximately half of the respondents never answered a private call (46.1%) or sent a message (51.9%) whilst in the presence of a patient, and about 40.0% indicated that they did occasionally answer a private call (43.0%) or send a message (41.1%).

More than one-third (36.7%) of respondents agreed that smart devices threaten patient confidentiality, but the majority (58.8%) did not agree that these devices hinder patient communication (Table 4). The majority of respondents felt that these devices improved both personal performance (69.2%) and patient care (79.0%). Whilst the highest percentage of respondents (39.2%) agreed that using a smart device when around patients was disrespectful, only 10.8% agreed that using a smart devise for diagnosis was unprofessional.

**TABLE 4:** Perceptions and practices of medical practitioners on the work-related use of smart devices.

Statement	Agree		Neutral		Disagree	
	*n*	%	*n*	%	*n*	%
**Patient care**						
I think that smart devices improve patient care (*n* = 119)	94	79.0	19	16.0	6	5.0
I think smart devices are a vital component of patient care (*n* = 120)	66	55.0	40	33.3	14	11.7
**Performance**						
I believe that my device can improve my performance at work (*n* = 120)	83	69.2	29	24.2	8	6.6
I think devices have improved the performance of colleagues at work (*n* = 119)	56	47.1	41	34.4	22	18.5
**Hindrance**						
I think that my device hinders communication with patients (*n* = 119)	16	13.5	33	27.7	70	58.8
I think that the usage of smart devices can slow down doctors (*n* = 120)	23	19.2	43	35.8	54	45.0
**Professionalism**						
I think the usage of smart devices around patients is disrespectful (*n* = 120)	47	39.2	45	37.5	28	23.3
I think the usage of smart devices for diagnosis is unprofessional (*n* = 120)	13	10.8	25	20.8	82	68.4
**Confidentiality**						
I think smart devices threaten patient confidentiality (*n* = 120)	44	36.7	51	42.5	25	20.8
I believe the risk to expose patient information is minimal (*n* = 120)	32	26.7	47	39.1	41	34.2
**Information browsing**						
I only trust information from applications bought through an app store (*n* = 120)	29	24.2	60	50.0	31	25.8
I think that Google is a trustworthy browser to look up medical information (*n* = 120)	23	19.2	45	37.5	52	43.3
**Other**						
I believe that smart devices improve medical education (*n* = 119)	87	73.1	27	22.7	5	4.2
I think that hospitals should provide smart devices (*n* = 120)	53	44.2	38	31.7	29	24.1

## Discussion

All of the medical practitioners at UAH brought a smart device, mostly their cell phone, to work. Work-related usage mainly included communication with colleagues and storage and usage of medical books. The most common applications on their smart devices were drug referencing and medical textbook applications.

Statistically, the age group 40–65 years had a significantly smaller percentage of medical practitioners with drug reference applications as compared with the age group 21–39 years. We hypothesised that this could be because the younger doctors had lesser knowledge regarding drugs and therefore used the applications to ascertain their prescription. Another reason could be that the older generation preferred to use medical formularies than using electronic drug referencing. There was also a general decrease in the number of applications uploaded from the age group 21–39 years to that of 40–65 years. Thus, we conclude from this trend that younger medical practitioners preferred to keep various applications pertaining to healthcare on their devices as opposed to their older counterparts.

In our study, 86.8% (including always, sometimes and occasionally) of the medical practitioners indicated that they send medical-related images of patients to their colleagues through WhatsApp for a second opinion. This is in line with a study^18^ conducted at a public hospital in Malaysia, concluding that 74.3% of health professionals used WhatsApp to obtain second opinion from their colleagues. Research has highlighted the ethical and legal issues raised by this ease of electronic capturing and transfer of patient images.^19^

In our study, nearly one-fifth of medical practitioners stated that they searched for images of their patients on social media. Furthermore, a few indicated that they placed images of patients on social media platforms. This was an unexpected finding because of the ethical and legal implications regarding this action, especially if it was without patient consent.

In terms of using secondary sources to assist in obtaining a diagnosis, nearly one-fifth of the respondents always used medical applications, with less than half of this proportion indicating that they always used Google as a source of reference. One of the reasons why the use of applications was preferred over Google could be because of an increase in the number of healthcare applications as well as their accuracy in recent years.^1^ Only 19.2% of our respondents agreed that Google is always a trustworthy platform for obtaining medical information; this might explain why few medical practitioners use Google.

Only 5.4% of the medical practitioners indicated that they never searched drug dosages or drug interactions on their smart devices. When compared with a study^16^ conducted on junior doctors in the United Kingdom, 48% indicated that they never used their smart devices for drug referencing. This could be because the study involved only junior doctors, who are equivalent to interns in South Africa, whereas our study included interns, registrars and consultants. Furthermore, a systematic review^3^ of the available medical-related applications showed that drug reference applications were one of the most widely available applications, and many are free, up-to-date and accurate.

A total of 89% of respondents said that they never (46.1%) or only occasionally (43.0%) answered private phone calls in the presence of their patients. This could indicate that most medical practitioners still found it disrespectful to answer private calls in the presence of patients unless it is an emergency. A study^20^ that investigated the use of cell phones by nurses has shown that 78.1% used their cell phones for private communication whilst at work. It could be hypothesised that different healthcare workers have different views when it comes to using their smart devices for private communication. Further studies will need to be conducted to assess whether or not this has an impact on patient safety and satisfaction.

Only 7.6% of medical practitioners indicated that they always used their smart devices for emergency care. In a study^21^ on smart devices as a health risk in critical care, 95% of the 40 anaesthetists whose hands were tested showed bacterial contamination after the use of a cell phone. Therefore, medical practitioners are to avoid the use of their smart devices in an emergency to prevent bacterial contamination of patients.^22^ Another reason could be the lack of time in an emergency to refer to one’s smart device to find relevant information about the situation at hand.

A quarter of medical practitioners in this study used their smart devices for patient communication. Even though the vast majority agreed that smart devices could improve patient care, just over half felt that it was vital for patient care. Therefore, it seems that the medical practitioners of this study did not consider communication with their patients through smart devices a crucial element regarding patient care.

In terms of confidentiality, 37.7% agreed that smart devices threaten it, whilst the majority (42.5%) took a neutral position. This showed the controversy over the usage of smart devices within the clinical environment. A study conducted at the University of Toronto has shown that 68% of the students believed that the usage of their smartphones posed a risk to patient confidentiality.^3^ Our percentage was much lower, possibly because either our practitioners were not yet aware of the implications thereof or the difference in terms of law enforcement to protect confidentiality. Other studies have also stated concerns regarding the legal issues over the use of smart devices, but solutions are still needed in this matter.^8,9,12^

If more medical practitioners feel the same way as the 68.4% of medical practitioners at UAH who indicated that the use of smart devices in diagnosis was not unprofessional, then smart devices would eventually play an important role in the assistance of obtaining a clinical diagnosis. This is also supported by the fact that the respondents of the study agreed that smart devices improved personal and colleagues’ performance. Based on the findings in a qualitative study of smartphone and tablet usage by house officers at a Ghana teaching hospital, Barnor-Ahiakor^17^ calls for the integration of such usage in medical curricula. Opperman and Janse van Vuuren^23^ have made a similar call with specific reference to the usage of WhatsApp and have, in addition, indicated that the Health Professions Council of South Africa (HPCSA) as a professional body should provide guidance regarding medical usage of social media. The need for institutional policies has been raised as well.^1^

### Limitations

Results reflect only the practices and opinions of medical practitioners from the largest departments and those who attended the selected weekly departmental or monthly morbidity and mortality meetings and chose to answer the questionnaire.

At some meetings, the questionnaires could be collected only after the meeting was over or on the next day because of the urgency of the meetings. This resulted in low response rates as we struggled to collect questionnaires. We could only compare responses from the Departments of Internal Medicine and Surgery because of the low number of responses from other departments.

## Conclusion

Medical practitioners at UAH all used smart devices at their workplace. The most common application used by medical practitioners was that of drug referencing. The medical practitioners found smart devices useful in their professional lives and felt that these improved their performance, although they had varied opinions about the ethical issues pertaining to smart devices and patient care. The younger generation made use of their smart devices more at work compared with the older generation. Thus, as more medical professionals start using smart devices in the medical environment, integration of such usage in medical curricula, discussion on professionalism, ethics and confidentiality in this context, and guidance from institutions and professional bodies become necessary.
